# Genetic analysis of products of conception using a HLPA/SNP-array strategy

**DOI:** 10.1186/s13039-019-0452-2

**Published:** 2019-09-02

**Authors:** Jun Mao, Huiling Wang, Haibo Li, Xiaoyan Song, Ting Wang, Jingjing Xiang, Hong Li

**Affiliations:** 1 Center for Reproduction and Genetics, The Affiliated Suzhou Hospital of Nanjing Medical University, Suzhou, Jiangsu China; 2 Center for Reproduction and Genetics, Suzhou Municipal Hospital, Suzhou, Jiangsu China; 3 Department of Gynaecology, The Affiliated Suzhou Hospital of Nanjing Medical University, Suzhou, Jiangsu China; 4 Ningbo Municipal Key Laboratory of Comprehensive Prevention and Treatment of Birth Defects, Ningbo Women & Children’s Hospital, Ningbo, Zhejiang, China

**Keywords:** HLPA, SNP-array, products of conception, chromosomal abnormality, miscarriage

## Abstract

**Background:**

Fetal chromosomal abnormalities was the most frequent cause of miscarriage, and the traditional testing method G-banded karyotyping has limitations. Then high-throughput ligation-dependent probe amplification (HLPA) and single nucleotide polymorphism array (SNP-array) were introduced for genetic analysis on products of conception (POC).

**Methods:**

HLPA and SNP-array analysis were combined. POC samples were initially tested using HLPA, followed by SNP-array analysis on samples that were found to be normal by HLPA.

**Results:**

Of the 326 POC samples tested, the overall abnormality rate was 54.6% (178/326), including 44.8% (146/326) chromosomal abnormalities identified by HLPA and 9.8% (32/326) additional chromosomal abnormalities further detected by SNP-array.

**Conclusions:**

The combination of HLPA and SNP-array analysis is an efficient and cost-effective strategy for genetic analysis of POC.

## Background

Miscarriage is defined as the spontaneous loss of pregnancy before 24 weeks of gestation, and 10-15% of clinically recognized pregnancies end in miscarriage [1]. Multiple factors are associated with miscarriage, including genetic, structural, infective, endocrine, immune factors and so on [2]. Among these, fetal chromosomal anomalies was the most frequent cause, which accounts for more than 50% of first-trimester miscarriage [3].

G-banded karyotyping is the conventional cytogenetic technique used in analysis of products of conception (POC), which could detect chromosomal aneuploidies, structural abnormalities, duplications or deletions (>5-10Mb), polyploidies and mosaicism. However, it has several limitations such as low resolution, high rates of culture failure and long reporting time [4]. Then some rapid techniques for genetic testing of chromosomal aneuploidies have emerged including quantitative fluorescent PCR (QF-PCR), fluorescence in situ hybridization (FISH), BACs-on-Beads (BOBs) and multiple ligation-dependent probe amplification (MLPA), but these techniques also have drawbacks such as restricted coverage and resolution on the whole genome due to limited number of chromosomal probes [5]. The advent of chromosomal microarray analysis (CMA) and next generation sequencing (NGS) enable us to identify submicroscopic imbalances on the whole genome with higher resolution [6, 7]. CMA includes array-based comparative genomic hybridization (aCGH) and single nucleotide polymorphism array (SNP-array) , and it is considered to be the fist-tier testing for detection of copy number variations (CNVs)[8].

In 2016, a new approach called high-throughput ligation-dependent probe amplification (HLPA) was established for genetic analysis of POC, which has been proved to be a rapid and accurate method for aneuploidy screening of 24 chromosomes in spontaneous abortion specimens [9, 10]. In this study, a HLPA/SNP-array strategy was applied to detect chromosomal abnormalities in POC, and the results indicated that the HLPA/SNP-array strategy was an efficient and economic method with improved diagnostic yield.

## Methods

### Samples

This study was approved by the institutional ethics committee of the Affiliated Suzhou Hospital of Nanjing Medical University, and written informed consent were obtained from all participants in the study. A total of three hundred and twenty six specimens of spontaneous abortion including chorionic villi and fetal tissues were collected at the Center for Reproduction and Genetics, the Affiliated Suzhou Hospital of Nanjing Medical University, Suzhou, Jiangsu, China, and the mean gestational age was 9.4 weeks (range: 5.1-17.1 weeks). The received POC samples were rinsed by saline solution immediately, and chorionic villi were separated using needles under a dissecting microscope. Then genomic DNA was extracted by QIAamp DNA Mini Kit (Qiagen GmbH, Hilden, Germany), and maternal cell contamination were ruled out for all the 326 samples by short tandem repeat (STR) profiling using Microreader™ 21 (Direct) ID System (Microread, Suzhou, China), which was used to simultaneously amplify 20 STR loci and the amelogenin gender marker.

### HLPA assay

HLPA assay was conducted using a Human 24 Chromosomes Aneuploidy Detection Kit (N9002, Genesky, Suzhou, China) according to the manufacturer’s instructions. Briefly, 200 ng of genomic DNA was added into a ligation premix for ligation reaction, then the ligation products were amplified by PCR reaction. PCR products were diluted and analyzed by capillary electrophoresis using an ABI 3130 Genetic Analyzer (Applied Biosystems, Foster City, CA, USA). Data analysis was performed by GeneMapper 5 (Applied Biosystems, Foster City, CA, USA) and CNV Reader 1.0 (Genesky, Suzhou, China). The CNV value of each target was calculated with a cut-off value 0.8-1.2 for one copy, 1.6-2.3 for two copies, and 2.5-3.5 for three copies. The CNV values of all probes on a chromosome between 2.5 to 3.5 suggest trisomy, while the CNV values of all probes on a chromosome in the range of 0.8 to 1.2 indicates monosomy. The CNV values of at least three consecutive probes on a chromosome within 2.5-3.5 or 0.8-1.2 may suggest partial duplication or partial deletion on the chromosome, and CNV values of at least three consecutive probes on a chromosome within 2.3-2.5 or 1.3-1.5 may indicate mosaicism or contamination.

### SNP-array analysis

The SNP-array analysis was performed on the Affymetrix CytoScan platform (Affymetrix, Santa Clara, CA, USA) following the manufacturer’s protocol. In brief, 250 ng of genomic DNA was digested, ligated, PCR amplified, purified, fragmented, labelled and hybridized to the Affymetrix 750K array, which includes 550,000 CNV markers and 200,000 SNP markers. After washing, staining and scanning of arrays, raw data were analyzed by Chromosome Analysis Suite (ChAS) 3.2 (Affymetrix, Santa Clara, CA, USA). CNVs were called at an minimum length of 50 kb containing at least 20 contiguous markers, and interpreted according to the standards and guidelines for interpretation and reporting of postnatal constitutional copy number variants released by the American College of Medical Genetics [11].

## Results

**Fig. 1:**
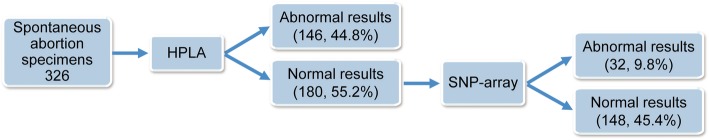
The analysis strategy of spontaneous abortion specimens

No maternal cell contamination was detected in all 326 POC samples. The analysis strategy used in this study was summarized in Fig. 1. A total of 326 spontaneous abortion specimens were successfully tested using HPLA, and 146 samples of which yielded abnormal results (146/326, 44.8%). The remaining 180 samples with normal HPLA results were further analyzed by SNP-array, and abnormal results were observed in 32 cases (32/326, 9.8%).

### Abnormalities identified by HPLA

**Fig. 2:**
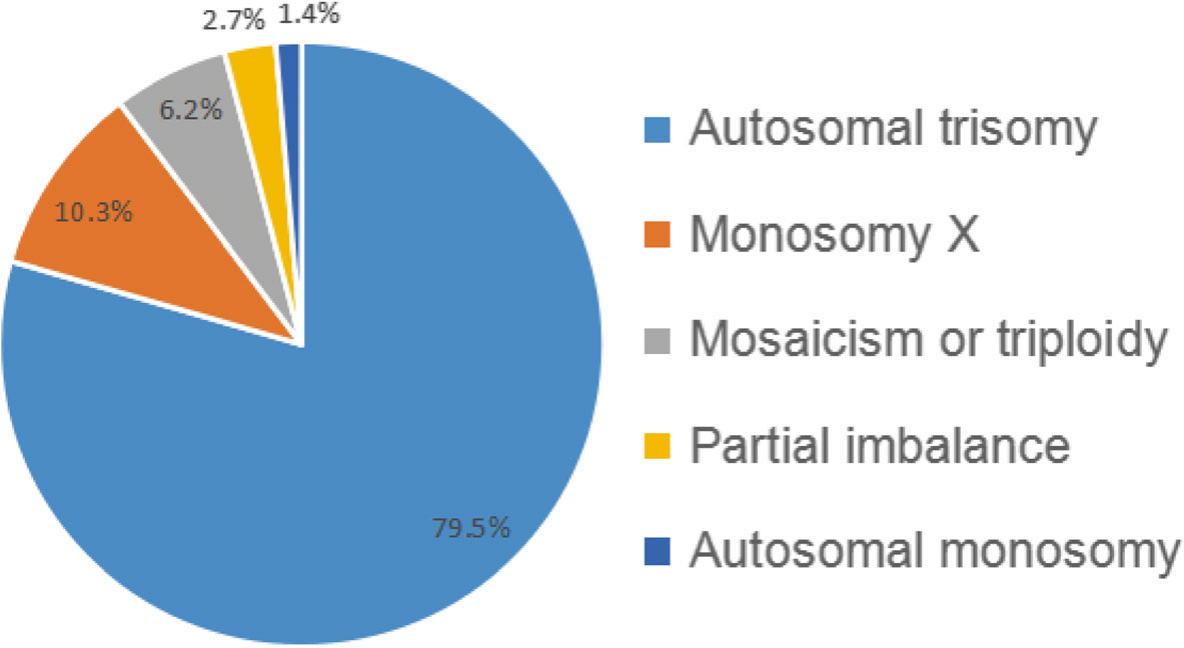
Abnormal results of HPLA

**Fig. 3:**
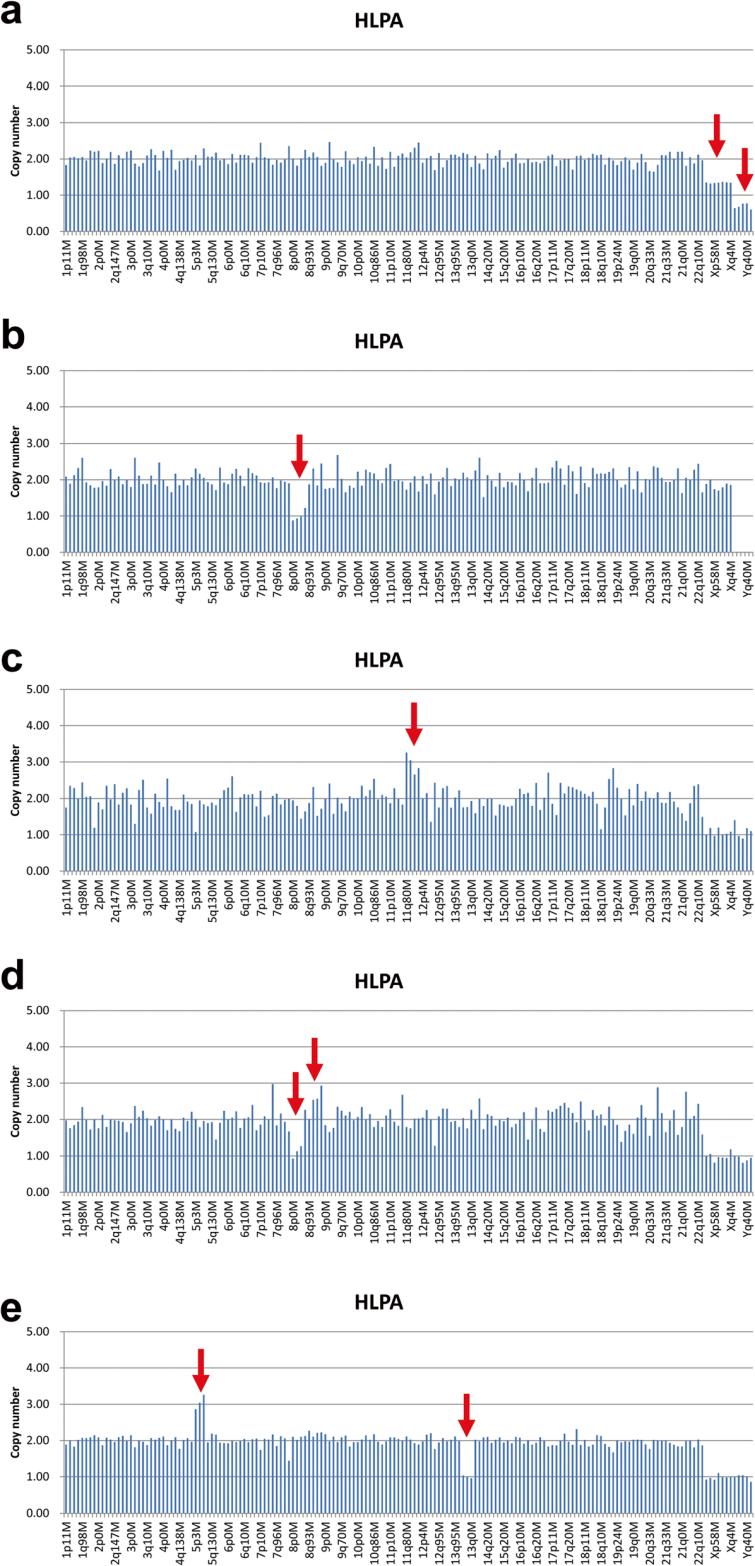
Copy number variation measurements of five POC samples by HPLA. Chromosomal loci for probes were displayed in the x-axis, and y-axis showed the calculated copy number. CNVs were indicated by red arrows. (**a**) genetic mosaic 46,XX/46,XY or 69, XXY triploidy; (**b**) deletion of 8p; (**c**) duplication of 11q; (**d**) partial deletion of 8p and partial duplication of 8q; (**e**) partial duplication of 5p and partial deletion of 13q

The results of HPLA revealed that 146 of 326 samples (44.8%) had chromosomal abnormalities. Among these, autosomal trisomy accounts for the largest proportion (116/146, 79.5%), followed by monosomy X (15/146, 10.3%), mosaicism or triploidy (9/146, 6.2%), partial imbalance (4/146, 2.7%), and autosomal monosomy (2/146, 1.4%) (Fig. 2). As shown in Fig. 3a, the HPLA results of 9 cases indicated that the CNV values of probes on X-chromosome were between 1.3-1.5, the CNV values of probes on Y-chromosome were between 0.6-0.8, and the ratio of CNV values of probes on autosomes, X-chromosome and Y-chromosome was about 3:2:1. Therefore, these 9 cases might be genetic mosaic 46,XX/46,XY or 69, XXY triploidy. 4 cases were found to have partial imbalance including deletion of 8p, duplication of 11q, partial deletion of 8p and partial duplication of 8q, and partial duplication of 5p and partial deletion of 13q (Fig. 3b-e). 2 cases with autosomal monosomy were monosomy 21.

### Abnormalities detected by SNP-array

**Fig. 4:**
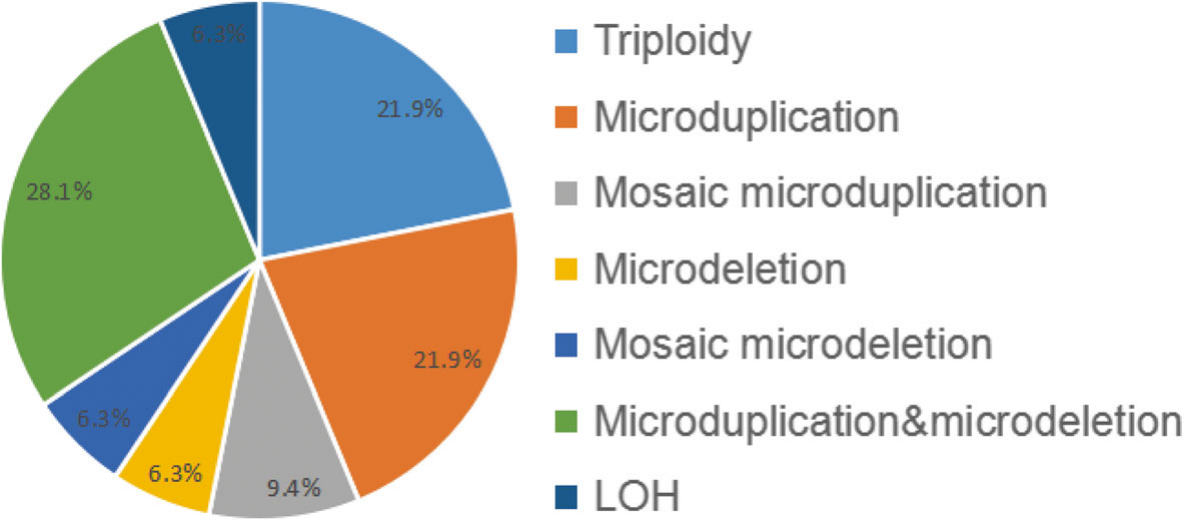
Abnormal results of SNP-array

One hundred eighty samples of spontaneous abortion tested to be normal by HPLA were further analyzed by SNP-array, and 32 samples (32/326, 9.8%) yielded abnormal results, including CNV, triploidy and loss of heterozygosity (LOH). Among these, CNV was the most common abnormality (23/32, 71.8%), which was divided into five subgroups: microduplication (7/32, 21.8%), mosaic microduplication (3/32, 9.4%), microdeletion (2/32, 6.25%), mosaic microdeletion (2/32, 6.25%) and microduplication&microdeletion (9/32, 28.1%) (Fig. 4, Table 1). 7 cases were found to be 69, XXX triploidy, accounting for 21.8% of all the abnormalities. And 2 cases (2/32, 6.25%) were tested to have LOH, including paternal uniparental disomy (UPD) of chromosome 7 and 27.532Mbp LOH at 5p15.33p14.1 containing the key region of Cri du Chat Syndrome. In addition, 9 samples tested to be genetic mosaic 46,XX/46,XY or 69, XXY triploidy by HPLA were also analyzed by SNP-array, and the results indicated that all these samples were 69, XXY triploidy. And 4 cases reported to have partial imbalance by HPLA were confirmed by SNP-array.

**Table 1:** CNVs identified by SNP-array in spontaneous abortions

No.	SNP-array result	Type	Interpretation
1	2p25.3p24.1 (2,506,047-23,993,512)x4, 21.49Mb; 2p25.3 (12,770-2,500,096)x1, 2.49Mb; 17q21.2q25.3 (39,043,284-81,041,823)x2-3; 42Mb, 30%.	Mosaic duplication, duplication&deletion	Pathogenic
2	10q26.11q26.3 (121,370,630-135,426,836)x3, 14.06Mb; 12q24.33 (131,262,269-133,257,821)x3, 1.99Mb.	Duplication	Pathogenic
3	Xq13.1q21.31 (71,514,629-88,581,567)x1-2, 17Mb, 22%.	Mosaic deletion	VOUS
4	19p13.3p12(260,911-22,318,810)x1-2, 22Mb, 27%.	Mosaic deletion	VOUS
5	6q23.2q27(133,299,322-170,914,297)x1-2, 37.62Mb, 53%; 6q25.3 (159,279,821-160,261,361)x3, 0.98Mb; 6q25.3q27(160,264,660-164,813,573)x1, 4.55Mb; 6q27(164,836,468-166,972,621)x3, 2.14Mb; 6q27(166,973,398-170,914,297)x1, 3.94Mb.	Mosaic deletion, duplication&deletion	Pathogenic
6	15q25.1q26.3 (79,052,984-102,429,040)x2-3, 23.38Mb, 32%	Mosaic duplication	VOUS
7	7q33q36.3 (134,801,449-159,119,707)x1, 24.32Mb, 7q terminal deletion syndrome; 11q21q25(96,159,484-134,937,416)x3, 38.78Mb, 11q terminal duplication syndrome.	Duplication&deletion	Pathogenic
8	19q12(28,870,035-29,879,116)x4, 1.00Mb	Duplication	Likely benign, maternal origin
9	7q36.3 (155,329,202-157,529,779)x4, 2.20Mb; 7q36.3 (157,536,855-158,879,019)x1, 1.34Mb; 19p13.3p12(260,911-58,956,816)x1-2, chr19, 34%.	Mosaic deletion, duplication&deletion	Pathogenic, de novo
10	Xp22.31 (7,143,928-7,690,002)x1, 0.55Mb	Deletion	Likely benign
11	18p11.21 (13,800,292-14,348,385)x3, 0.55Mb	Duplication	Likely benign
12	9p24.3p21.1 (208,454-28,535,563)x2-3, 28.32Mb, 58%; 18q21.31q23(54,265,846-78,013,728)x1, 23.74Mb; 18q21.2q21.31 (50,986,553-54,069,099)x3, 3.08Mb	Mosaic duplication, duplication&deletion	Pathogenic
13	4q31.23q35.2 (149,107,084-190,957,460)x3, 41.85Mb; 7q34q36.3 (143,084,599-159,119,707)x1, 16.04Mb	Duplication&deletion	Pathogenic
14	Xp22.31 (6,455,150-8,135,568)x2, 1.68Mb.	Duplication	Likely benign, maternal origin
15	1p22.2 (89,523,245-91,159,433)x1, 1.64Mb.	Deletion	VOUS
16	7q22.1q36.3 (101,562,358-159,119,707)x2-3, 57.56Mb, 37%.	Mosaic duplication	VOUS
17	5p15.33p13.3 (113,576-29,104,386)x3, 28.99Mb; 13q22.3q34(77,782,493-115,011,636)x1, 37.23Mb.	Duplication&deletion	Pathogenic
18	2q37.3 (238,138,565-238,616,893)x3, 0.48Mb.	Duplication	Likely benign
19	12q24.23 (119,276,763-119,682,258)x3, 0.41Mb.	Duplication	VOUS
20	20p12.3p12.2 (8,633,732-9,375,262)x3, 0.74Mb.	Duplication	VOUS
21	2q37.1q37.3 (235,368,568-237,312,027)x4, 1.94Mb; 2q37.3 (238,781,791-242,782,258)x1, 4Mb.	Duplication&deletion	Pathogenic
22	18q22.2q23(68,019,984-78,013,728)x1, 9.99Mb; 3q27.3q29(186,570,452-195,835,968)x3, 9.27Mb.	Duplication&deletion	Pathogenic
23	20p13q13.33 (61,661-62,913,645)x2-3, 62.85Mb, 31%.	Mosaic duplication	Pathogenic

## Discussion

Elucidating the etiology of miscarriage is important for genetic consultation and management of the couple’s future pregnancies. Although the causes of miscarriage could be complicated, genetic abnormalities, mainly aneuploidies, was the most frequent cause of first-trimester pregnancy loss, which are detected in 45-70% of sporadic miscarriages and 25-57% of recurrent miscarriages [12]. Thus exploring the genetic cause of pregnancy loss is of great importance.

HLPA is a method modified from MLPA to detect the copy number of 24 chromosomes by analyzing 170 genomic loci in one reaction [9, 10]. In addition to aneuploidy, SNP-array can also detect CNVs, LOH, polyploidy and mosaicism at the genome-wide level. However, balanced chromosomal translocation and low-level mosaicism (<10-15%) could not be identified by SNP-array. The turnaround time of HPLA is within 24h, which is shorter than that of SNP-array (2-3d) and conventional karyotyping (21-30d). The cost of HPLA is comparable to that of conventional karyotyping and much lower than that of SNP-array (1/10 of the cost of SNP-array). And in our institution, the failure rates of genetic analysis of POC by HPLA, SNP-array and conventional karyotyping are 2.1%, 1% and 35.6% respectively (unpublished data). Here we report an efficient and cost-effective HLPA/SNP-array strategy for genetic analysis of POC. Our analysis results of 326 POC specimens using this strategy indicated that the overall abnormality rate was 54.6%. Among these, 44.8% of samples were found to be abnormal by HLPA and SNP-array detected 9.8% additional chromosomal abnormalities, which is consistent with a previous report that CMA identified 13% (95% CI 8.0–21.0) additional chromosome aberrations over conventional karyotyping [13]. And the expense of SNP-array analysis is avoided for 40.8% of samples found to be aneuploid by HPLA. Hence the combination of HLPA and SNP-array in genetic analysis of POC is more cost-effective than testing by SNP-array alone.

Previous studies revealed that submicroscopic CNVs could be one of genetic causes in pregnancy losses [14–19].In this study, 23 CNVs were identified in 326 POC samples using SNP-array, of which 11 CNVs were interpreted as pathogenic for these CNVs were cytogenetically visible alterations (>3-5 Mb) without well-established cytogenetic heteromorphisms, and 7 CNVs were classified as VOUS because whether these CNVs cause spontaneous abortions is still an open question (Table 1). Moreover, LOH and triploidy were detected in 9 cases by SNP-array, which were tested to be normal by HPLA. In addition, 4 POC samples found to have partial imbalance by HPLA were confirmed by SNP-array, indicating that HPLA can also detect microduplications and microdeletions [9, 10].

## Conclusions

A combined HLPA and SNP-array analysis is an efficient and cost-effective strategy for genetic analysis of POC. We recommend the use of HLPA for initial genetic screening on POC, and subsequent SNP-array analysis on POC with normal HLPA results.

## Data Availability

All data are available upon request.

## Abbreviations

aCGH: Array-based comparative genomic hybridization
BOBs: BACs-on-Beads
CMA: Chromosomal microarray analysis
CNVs: Copy number variations
FISH: Fluorescence in situ hybridization
HLPA: High-throughput ligation-dependent probe amplification
LOH: Loss of heterozygosity
MLPA: Multiple ligation-dependent probe amplification
NGS: Next generation sequencing
POC: Products of conception
QF-PCR: Quantitative fluorescent PCR
SNP-array: Single nucleotide polymorphism array
STR: Short tandem repeat
UPD: Uniparental disomy
